# Regulation of testosterone synthesis in Leydig cells by ClC-2 chloride channel

**DOI:** 10.1530/REP-24-0432

**Published:** 2025-07-18

**Authors:** Ssu-Ju Fu, Min-Shan Syu, Chih-Yu Tang, Ching-Yuan Huang, Chung-Jiuan Jeng, Chih-Yung Tang, Meng-Chun Hu

**Affiliations:** ^1^ Graduate Institute of Physiology, National Taiwan University College of Medicine, Taipei, Taiwan; ^2^ Institute of Anatomy and Cell Biology, College of Medicine, National Yang Ming Chiao Tung University, Taipei, Taiwan; ^3^ Brain Research Center, National Yang Ming Chiao Tung University, Taipei, Taiwan

**Keywords:** steroidogenesis, Leydig MA-10 cells, Cyp17a1, testicular testosterone, shRNA knockdown

## Abstract

**In brief:**

Leydig cell steroidogenesis is essential for spermatogenesis and male fertility. This research reveals that ClC-2 chloride channels enhance testosterone production via increased expression of steroidogenic genes in Leydig cells.

**Abstract:**

Testicular Leydig cells are the main source of testosterone, which is essential for spermatogenesis and male fertility. Leydig cell steroidogenesis is regulated by chloride channels, the molecular composition of which remains obscure. ClC-2 is a hyperpolarization-activated chloride channel present in virtually all tissues. ClC-2 deficiency is associated with the loss of male germ cells in mice and male azoospermia in humans. However, the functional significance of ClC-2 in Leydig cells is still unclear. Herein, we aimed to test the hypothesis that the ClC-2 chloride channel may play a regulatory role in Leydig cell steroidogenesis. ClC-2 expression was validated in testicular Leydig cells in adult mice. Lentivirus-delivered shRNA knockdown of endogenous ClC-2 expression in the mouse Leydig tumor cell line MA-10 significantly reduced basal mRNA expression of steroidogenic genes involved in testosterone synthesis, resulting in a dramatic decline in basal testosterone secretion. Application of 8-bromo-cAMP effectively increased the expression of steroidogenic genes and production of testosterone in MA-10 cells. These 8-bromo-cAMP–induced effects were notably attenuated following ClC-2 knockdown, as well as inhibition of the T-type voltage-gated calcium channel. Conversely, enhanced ClC-2 protein level promoted steroidogenic gene expression and testosterone synthesis in MA-10 cells. Importantly, *in vivo* intratesticular injection of a shClC-2 lentivirus potently diminished intratesticular testosterone levels in adult mice. Taken together, our data highlight a critical role of ClC-2 in regulating testosterone production in Leydig cells and a mechanistic link between testosterone deficiency and loss-of-function mutation in the ClC-2 chloride channel.

## Introduction

Spermatogenesis is a complex developmental process by which sperm are produced in the seminiferous tubules of the testes (Xiao *et al.* 2014). Sertoli cells, the supportive cells in the seminiferous tubules, provide both physical and nutritional assistance for the development and maturation of male germ cells (Cheng & Mruk 2012, Ni *et al.* 2019). The blood-testis barrier is created by adjacent Sertoli cells, which divide the seminiferous epithelium into basal and adluminal compartments. This division establishes a specialized microenvironment crucial for the development and maturation of both meiotic and postmeiotic germ cells (Cheng & Mruk 2012, Mruk & Cheng 2015). Spermatogenesis is tightly regulated by various hormonal and cellular mechanisms to ensure the continuous production of mature sperm cells. Testosterone is an androgen produced in the testes that is required for the maintenance of spermatogenesis (Smith & Walker 2014). The androgen receptor (AR) is present in Sertoli cells and functions as a major mediator for the testosterone control of germ cell development. Mice lacking the AR in Sertoli cells showed infertility because spermatogenesis was blocked in meiosis (Chang *et al.* 2004, De Gendt *et al.* 2004). In addition, the formation of the Sertoli cell barrier was impaired in these mutant mice (Willems *et al.* 2010).

Leydig cells, located in the interstitial space of testes, are responsible for the production and secretion of the essential male sex hormone testosterone (Saez 1994). Like all steroid hormones, testosterone is synthesized from cholesterol through the steroid biosynthesis pathway (Miller & Auchus 2011). Testosterone production in Leydig cells is mainly regulated by luteinizing hormone (LH) through the second messenger cAMP (de Mattos *et al.* 2023). Steroidogenic acute regulatory (STAR) protein expression is rapidly increased by the stimulation of LH, which facilitates the translocation of cholesterol from the outer to the inner mitochondrial membrane (Clark *et al.* 1994). Cholesterol is then converted into pregnenolone by a cholesterol side-chain cleavage enzyme, P450scc (CYP11A1). Subsequently, 3β-hydroxysteroid dehydrogenase/isomerase 2 (HSD3B2), 17-α hydroxylase/17, 20 lyase (CYP17A1), and 17β-hydroxysteroid dehydrogenase (HSD17B) are involved in the production of testosterone (Zirkin & Papadopoulos 2018). In particular, CYP17A1 plays a significant role in catalyzing multiple key processes of androgen production (Yoshimoto & Auchus 2015).

Chloride channels have long been suggested to play an important role in Leydig cell steroidogenesis. In rat Leydig cells, for example, LH-induced steroidogenesis was inhibited by the chloride channel blocker 4-acetamido-4′-isothiocyanatostilbene-2,2′-disulfonic acid (SITS) (Choi & Cooke 1990). In addition, removal of extracellular chloride enhanced LH- or cAMP-stimulated steroidogenesis in both rat Leydig cells and the mouse Leydig tumor cell line MA-10 (Choi & Cooke 1990, Ramnath *et al.* 1997, Cooke *et al.* 1999). Nevertheless, the molecular nature of the chloride channel involved in the regulation of Leydig cell steroidogenesis remains unclear.

ClC-2 is a hyperpolarization-activated chloride channel expressed in virtually all tissues (Bi *et al.* 2014). Despite their potential effect in modulating chloride homeostasis and membrane potential, the precise physiological importance of ClC-2 channels remains largely obscure. Mice with complete ClC-2 knockout or homozygous ClC-2 truncation mutation exhibited a dramatic loss of retinal photoreceptors and male germ cells, leading to blindness and male infertility, respectively (Bosl *et al.* 2001, Nehrke *et al.* 2002, Edwards *et al.* 2010). In addition, the loss of ClC-2 in mice resulted in leukoencephalopathy, manifesting as fluid-filled vacuoles within myelin of central neurons (Blanz *et al.* 2007, Edwards *et al.* 2010). Mutations in the human *CLCN2* gene, which encodes the ClC-2 chloride channel, were first found in a type of leukoencephalopathy with intramyelinic edema (Depienne *et al.* 2013). Subsequently, a *CLCN2* missense mutation was reported in a man showing infertility and subclinical leukoencephalopathy, as evidenced by the presence of azoospermia in semen analyses (Di Bella *et al.* 2014). A recent study involving genetic screening in infertile men also linked a loss-of-function mutation in ClC-2 to azoospermia in a patient (Jeworutzki *et al.* 2021). Together, these studies imply that ClC-2 channels may play a critical role in spermatogenesis. We have previously found that ClC-2 is highly expressed in MA-10 cells and primary mouse Leydig cells (Fu *et al.* 2020, 2021). Nonetheless, the precise functional significance of ClC-2 in Leydig cells is still unknown. In this study, we aimed to explore the potential roles of ClC-2 in testosterone synthesis in Leydig cells.

## Materials and methods

### Cells and reagents

Mouse Leydig MA-10 cells were grown in Dulbecco’s Modified Eagle’s Medium/Nutrient Mixture F-12 (DMEM/F-12) supplemented with 10% fetal bovine serum (Biological Industries, Israel), 100 U/mL penicillin, and 100 μg/mL streptomycin (Thermo Fisher Scientific, USA) at 37°C in 5% CO_2_. 8-bromo-cAMP (8-Br-cAMP) was purchased from Selleck Chemicals (USA). 17-allylamino-17-demethoxygeldanamycin (17-AAG) (LC Laboratories, USA) and mibefradil (Sigma-Aldrich, USA) were dissolved in 0.1% dimethyl sulfoxide (DMSO) (Sigma-Aldrich).

### shRNA knockdown and generation of stable cell lines

The shRNA-expressing lentiviral plasmids (pLKO.1-shRNA) were purchased from the National RNAi Core Facility (Academia Sinica, Taiwan). Mouse *Clcn2* was targeted with the constructs TRCN0000422398 (shClC2-A) and TRCN000069380 (shClC2-Z), and the construct TRCN0000072223 (shLacZ), which is specific for LacZ, was used as a control. To produce lentiviral particles, the pLKO.1-shRNA construct was co-transfected with the packaging plasmid pCMV-ΔR8.91 and envelope plasmid pMD.G into HEK293T cells. The culture medium containing lentivirus was collected, filtered, and incubated with MA-10 cells in the presence of 8 μg/mL polybrene (Sigma-Aldrich). Cells expressing the shRNA were selected with medium containing 8 μg/mL puromycin (Sigma-Aldrich). The puromycin-resistant cells were further maintained in medium with 2 μg/mL puromycin.

### Immunofluorescence

Testes of 8-week-old C57BL/6J mice were fixed with 4% paraformaldehyde, dehydrated through a graded ethanol series (70, 80, 95, and 100%), cleared in xylene, and embedded in paraffin. Tissue sections of 5 μm were deparaffinized in xylene and rehydrated through a graded ethanol series (100, 95, 70%) to distilled water. Antigen retrieval was then performed in Tris–EDTA buffer (10 mM Tris, pH 9, 1 mM EDTA, 0.05% Tween-20) at 100°C for 30 min. After cooling and washing with PBS, slides were incubated in Blocking One Histo (Nacalai Tesque, Inc., Japan) blocking buffer for 20 min at room temperature. The slides were then incubated overnight at 4°C with mouse anti-ClC-2 (1:50; sc-377284 from Santa Cruz Biotechnology, USA) and rabbit anti-HSD3B1 (1:200; GTX102813 from GeneTex, USA) primary antibodies in diluted blocking buffer (0.1% Tween-20, 1:20 Blocking One Histo in PBS). The slides were washed three times in PBS, incubated with Alexa Fluor 488 goat anti-mouse (Thermo Fisher Scientific, USA) and Alexa Fluor 568 goat anti-rabbit (Thermo) secondary antibodies, and then washed with PBS. Subsequently, sections were treated with TrueBlack Plus Lipofuscin Autofluorescence Quencher buffer (1:80, from Biotium, USA) for 5 min at room temperature and then mounted using Vectashield HardSet Antifade Mounting Medium containing DAPI. Cells grown on coverslips were fixed with 4% paraformaldehyde in PBS at room temperature for 20 min. Immunofluorescence staining was performed as previously described (Fu *et al.* 2020). Fluorescence images were acquired using the Leica TCS SP8 laser-scanning confocal microscope (Germany). Optical sectioning was performed using the confocal z-stack mode with a step size of 1.1 μm to collect serial images through the entire thickness of the sample. Pinhole size was set to 1 Airy unit to ensure optimal axial resolution. Excitation and emission wavelengths were set according to the fluorophores used (i.e. Alexa Fluor 488, Alexa Fluor 568, and DAPI).

### Western blot analysis

Cells were lysed in RIPA buffer (50 mM Tris–HCl, pH 8, 150 mM NaCl, 1% NP-40, 0.5% sodium deoxycholate, 0.1% sodium dodecyl sulfate, 5 mM EDTA, 1 mM EGTA, 5 mM DTT, 2 mM phenylmethylsulfonyl fluoride, and 10 μg/mL leupeptin) and incubated on ice for 30 min. After centrifugation, the supernatant fraction was collected and subjected to western blot analysis. The antibodies used in this study included anti-ClC-2 (Alomone Labs, Israel) and anti–α-tubulin (Abcam, UK).

### RNA extraction and quantitative reverse transcription PCR (RT-qPCR)

Total RNA was extracted from cells using TOOLSmart RNA Extractor (Biotools, Taiwan) according to the manufacturer’s instructions. One microgram of total RNA was reverse-transcribed into cDNA with the Magic RT cDNA synthesis kit (Bio-Genesis Technologies, Taiwan) as described by the manufacturer. Quantitative PCR (qPCR) was conducted on a StepOnePlus machine (Thermo) using TOOLS 2X SYBR qPCR Mix (Biotools). The primer sequences used for the qPCR analysis are provided in Table 1. Relative mRNA levels were calculated using the 2^−ΔΔCt^ method and normalized to GAPDH.

**Table 1:** Primer sequences used for RT-qPCR.

Gene	Primer sequences (5’→3′)	
	Forward	Reverse
*Star*	GGA​AGT​CCC​TCC​AAG​ACT​AAA​C	ACT​CTA​TCT​GGG​TCT​GCG​ATA
*Cyp11a1*	ACA​TGG​CCA​AGA​TGG​TAC​AGT​TG	ACG​AAG​CAC​CAG​GTC​ATT​CAC
*Cyp17a1*	TGA​CCA​GTA​TGT​AGG​CTT​CAG​TCG	TCC​TTC​GGG​ATG​GCA​AAC​TCT​C
*Hsd3b1*	TGG​ACA​AAG​TAT​TCC​GAC​CAG​A	GGC​ACA​CTT​GCT​TGA​ACA​CAG
*Gapdh*	AAT​CCC​ATC​ACC​ATC​TTC​CA	TGG​ACT​CCA​CGA​CGT​ACT​CA

### Steroid measurement

Cells were cultured in serum-free medium treated with or without 0.5 mM 8-Br-cAMP for the indicated time. The medium was collected, and progesterone and testosterone concentrations were determined using the enzyme-linked immunosorbent assay (ELISA) (Cayman Chemical Company, USA).

The extraction of mouse intratesticular testosterone was based on previously described procedures (Jeyaraj *et al.* 2005). The samples were homogenized in 80 μL of PBS and then sonicated for 5 s three times with a 5 s interval. The sample was transferred to a 15 mL tube, combined with 5 mL of ether, and vortexed for 2 min. After a 10 min incubation at room temperature, the upper layer of ether was transferred to a new tube. The aqueous phase was combined with another 3 mL of ether and vortexed for 2 min, and the mixture was placed at −80°C for 15 min. The upper layer of ether was removed, and the pooled ether samples were dried by nitrogen gas. The samples were dissolved in 0.5 mL of PBS, vortexed for 2 min, and stored at −80°C. The testosterone concentration in the extract was measured with ELISA.

### Intratesticular injection assay

Animal experiments were approved by the Institutional Animal Care and Use Committee, NTU. All methods were performed in accordance with the relevant guidelines and regulations. The lentivirus stock for shClC2-A and shLacZ was prepared at a concentration of ∼3 × 10^9^ RIU/mL by the National RNAi Core Facility. The intratesticular injection of lentivirus was performed according to a previous study (Kojima *et al.* 2003). In brief, male 8-week-old C57BL/6 mice were anesthetized with pentobarbital, and a scrotal incision was made. Lentivirus solution (25 μL) was injected into the interstitial space of the right testis using a 30-gauge needle. After 7 days, animals were sacrificed, and the right testis was removed and cut into several pieces. Samples were frozen in liquid nitrogen and stored at −80°C.

### Statistical analysis

Numerical data were pooled from at least three independent experiments. Quantitative values are presented as mean ± SD. The statistical analysis was performed with Student’s *t*-test or one-way ANOVA, followed by the Bonferroni test. The data were analyzed using the GraphPad Prism 8 software (GraphPad Software). A difference was considered statistically significant at *P* < 0.05.

## Results

### Verification of endogenous ClC-2 expression in Leydig cells

**Figure 1:**
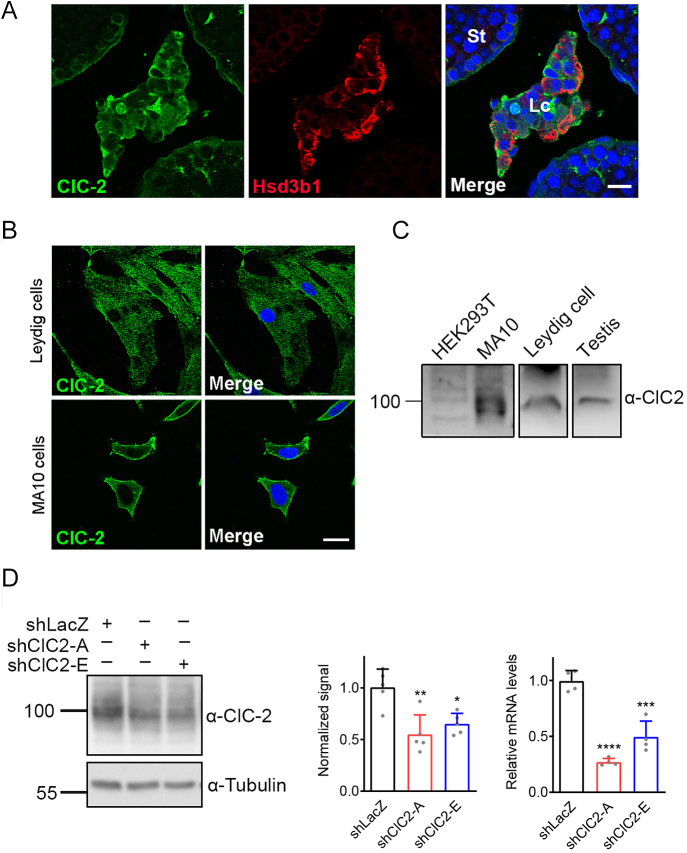
Expression of endogenous ClC-2 in Leydig cells. (A) Immunofluorescence staining of ClC-2 (green) and *Hsd3b1* (red) was performed on mouse testicular sections. *Hsd3b1* is a specific marker for Leydig cells. Nuclei were stained with DAPI (blue). St, seminiferous tubules; Lc, Leydig cells. Scale bar, 25 μm. See Supplementary Fig. 1 for additional images. (B) Immunofluorescence staining patterns of ClC-2 in cultured mouse Leydig cells and MA-10 cells. Scale bar, 25 μm. (C) Representative immunoblots showing ClC-2 expression in lysates prepared from mouse testes, cultured mouse Leydig cells, MA-10 cells, and HEK293T cells. (D) MA-10 cells were transduced with shLacZ (negative control), or two shRNAs specific for mouse ClC-2 (shClC2-A, shClC2-E). The shClC-2-mediated knockdown effects on ClC-2 protein expression were analyzed by immunoblotting (left panel) and quantified by standardizing with the corresponding α-tubulin levels (middle panel). Total RNA was extracted, and ClC-2 mRNA levels were measured by RT-qPCR (right panel). Protein and mRNA levels were normalized with respect to the corresponding shLacZ control. Normalized ClC-2 protein levels (*n* = 5): shLacZ, 1.01 ± 0.18; shClC2-A, 0.55 ± 0.19; shClC2-E, 0.65 ± 0.10. Relative ClC-2 mRNA levels (*n* = 4): shLacZ, 1.00 ± 0.09; shClC2-A, 0.27 ± 0.03; shClC2-E, 0.49 ± 0.14. Asterisks indicate statistically significant differences compared with the shLacZ control (*P < 0.05; **P < 0.01; ***P < 0.001; ****P < 0.0001). The original uncropped micrographs and immunoblots are presented in Supplementary Fig. 2.

We first examined the expression of ClC-2 in the testis interstitium of adult mice. Immunofluorescence staining revealed that ClC-2 was located on the plasma membrane of Leydig cells, represented with Hsd3b1, the steroidogenic enzyme known to be localized to the membranes of endoplasmic reticulum and mitochondria (Fig. 1A; Supplementary Figs 1 and 2 (see section on Supplementary materials given at the end of the article)) (Simard *et al.* 2005). ClC-2 was abundantly present in mouse primary Leydig cells and the mouse Leydig tumor cell line MA-10, as revealed by both immunofluorescence (Fig. 1B; Supplementary Fig. 2) and western blotting analysis (Fig. 1C; Supplementary Fig. 2). To further confirm the presence of ClC-2, we knocked down ClC-2 using a lentivirus delivering two ClC-2–specific short hairpin RNAs (shClC2-A and shClC2-E) in MA-10 cells and established stable shClC2 cells after selection by puromycin. shClC2 MA-10 cells had significantly lower protein and mRNA levels of ClC-2 compared to those in control shLacZ MA-10 cells (Fig. 1D). These data indicate that ClC-2 is prominently expressed in mouse Leydig cells.

### Reduction of testosterone synthesis in Leydig cells by ClC-2 knockdown

**Figure 2:**
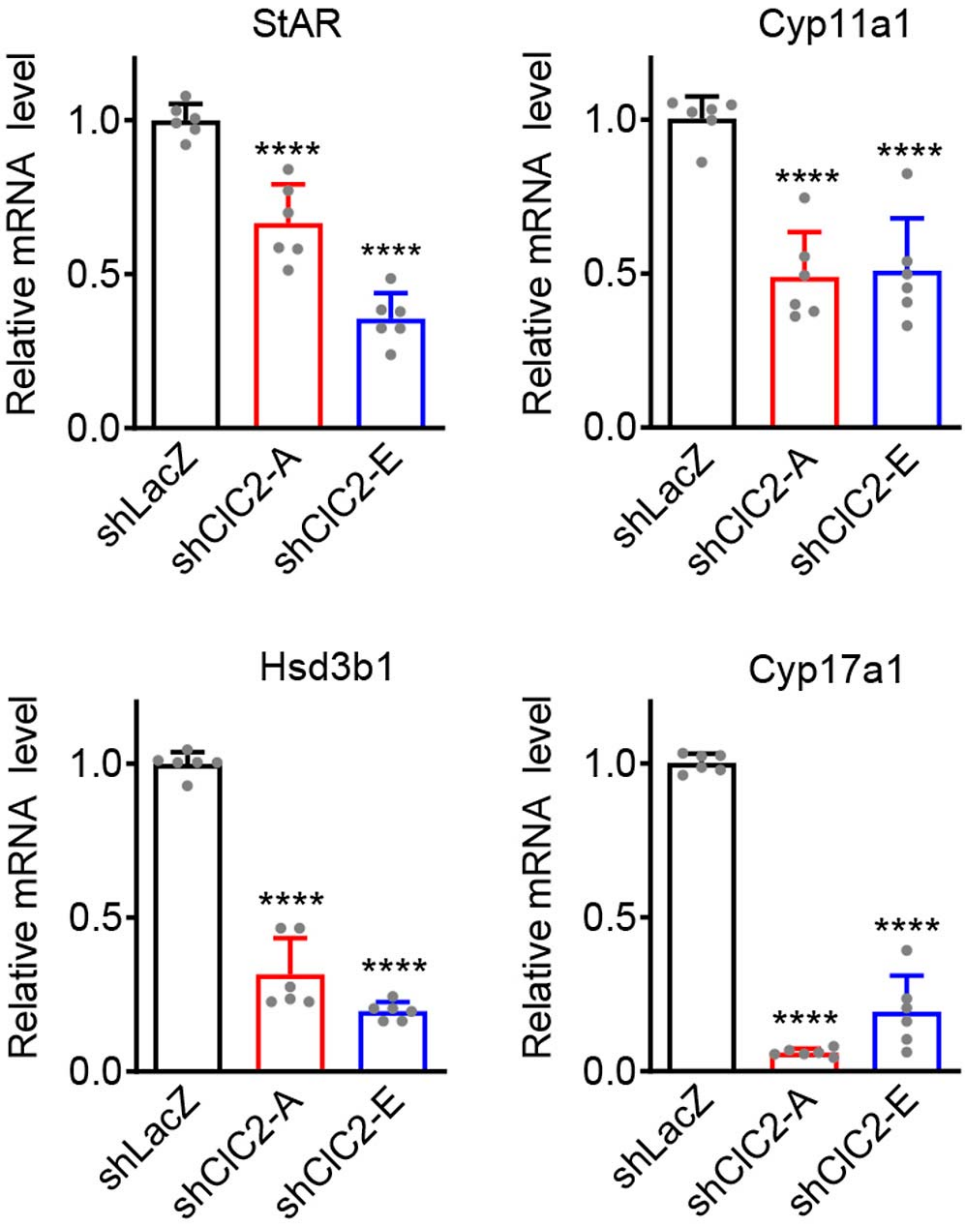
Attenuation of basal steroidogenic gene expression in Leydig cells by ClC-2 knockdown. The mRNA levels for the indicated steroidogenic genes in MA-10 cells stably expressing shClC-2 or control shLacZ were quantified by RT-qPCR, followed by normalization with respect to the corresponding shLacZ control. Relative *Star* mRNA levels (*n* = 6): shLacZ, 1.00 ± 0.05; shClC2-A, 0.66 ± 0.13; shClC2-E, 0.36 ± 0.08. Relative *Cyp11a1* mRNA levels (*n* = 6): shLacZ, 1.00 ± 0.07; shClC2-A, 0.49 ± 0.15; shClC2-E, 0.51 ± 0.17. Relative *Hsd3b1* mRNA levels (*n* = 6): shLacZ, 1.00 ± 0.04; shClC2-A, 0.32 ± 0.12; shClC2-E, 0.20 ± 0.03. Relative *Cyp17a1* mRNA levels (*n* = 6): shLacZ, 1.00 ± 0.03; shClC2-A, 0.06 ± 0.01; shClC2-E, 0.19 ± 0.12. Asterisks indicate statistically significant differences compared with the shLacZ control (****P < 0.0001).

**Figure 3:**
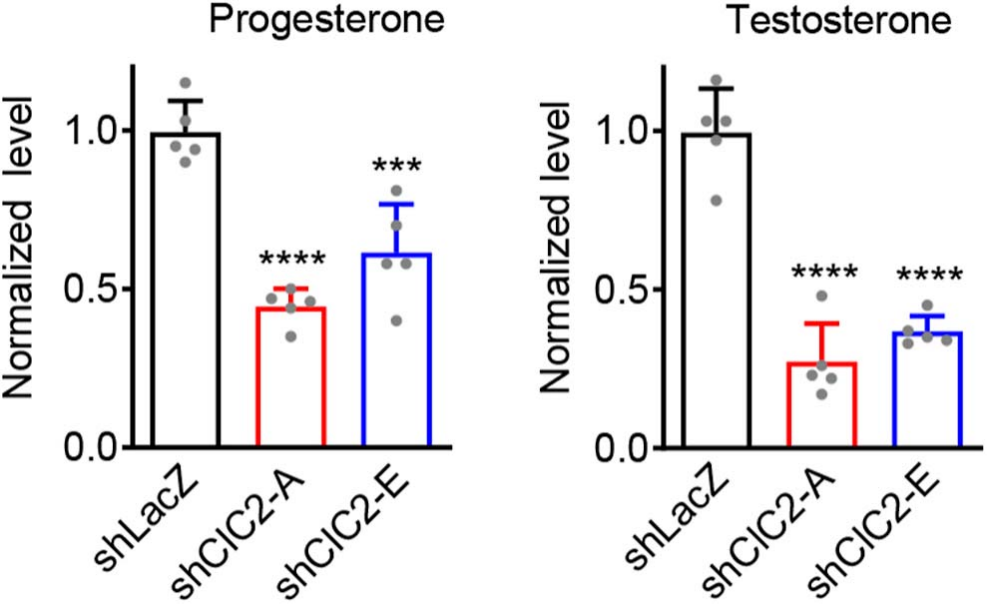
Decrease in basal steroid levels in Leydig cells by ClC-2 knockdown. MA-10 cells stably expressing shClC-2 or control shLacZ were cultured in serum-free medium for 24 h. The levels of progesterone or testosterone in the medium were measured by ELISA. Hormone levels were normalized with respect to the corresponding shLacZ control. Normalized progesterone levels (*n* = 5): shLacZ, 1.00 ± 0.10; shClC2-A, 0.44 ± 0.06; shClC2-E, 0.61 ± 0.15. Normalized testosterone levels (*n* = 5): shLacZ, 1.00 ± 0.14; shClC2-A, 0.27 ± 0.12; shClC2-E, 0.37 ± 0.05. Asterisks indicate statistically significant differences compared with the shLacZ control (***P < 0.001; ****P < 0.0001).

To investigate the roles of ClC-2 in Leydig cell steroidogenesis, we first evaluated the effects of ClC-2 knockdown on basal steroidogenic gene expression in MA-10 cells. Compared with control shLacZ MA-10 cells, the mRNA expression of steroidogenic genes involved in testosterone biosynthesis, including *Star*, *Cyp11a1*, *Hsd3b1*, and *Cyp17a1*, was reduced in shClC-2 MA-10 cells, with *Cyp17a1* impacted the most significantly (Fig. 2). Previous studies have shown that, under basal conditions, the main steroid produced by MA-10 cells is progesterone, while the amount of testosterone is very low (Ascoli 1981, Mellon & Vaisse 1989). Our ELISA assay revealed that basal levels of progesterone and testosterone production were significantly decreased in shClC-2 MA-10 cells compared with those in control shLacZ MA-10 cells (Fig. 3).

**Figure 4:**
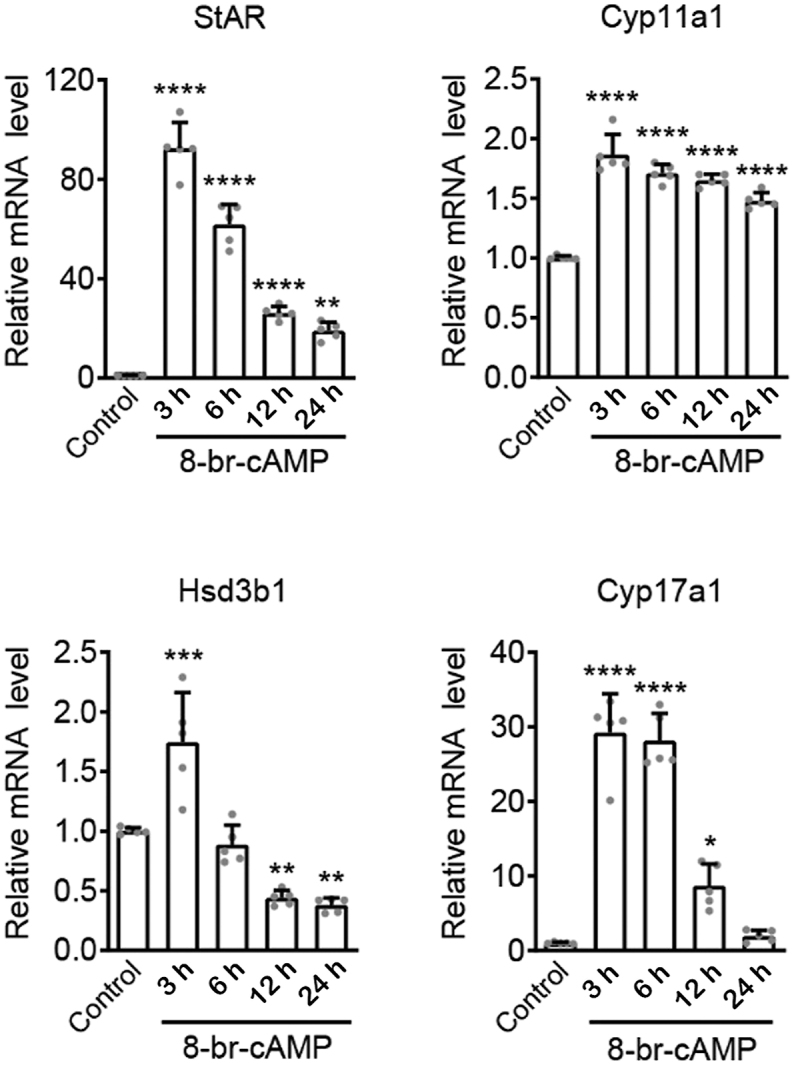
Promotion of steroidogenic gene expression in Leydig cells by 8-Br-cAMP. MA-10 cells were treated with 0.5 mM 8-Br-cAMP for 3, 6, 12, or 24 h. Cells with no 8-Br-cAMP treatment were used as the control. The mRNA levels for the indicated steroidogenic genes were quantified by RT-qPCR, followed by normalization with respect to the corresponding no-treatment control. Relative *Star* mRNA levels (*n* = 5): control, 1.00 ± 0.03; 3 h, 92.42 ± 10.41; 6 h, 61.78 ± 8.14; 12 h, 26.13 ± 2.74; 24 h, 19.01 ± 3.46. Relative *Cyp11a1* mRNA levels (*n* = 5): control, 1.00 ± 0.02; 3 h, 1.86 ± 0.17; 6 h, 1.71 ± 0.08; 12 h, 1.65 ± 0.05; 24 h, 1.48 ± 0.07. Relative *Hsd3b1* mRNA levels (*n* = 5): control, 1.00 ± 0.03; 3 h, 1.75 ± 0.42; 6 h, 0.89 ± 0.16; 12 h, 0.44 ± 0.06; 24 h, 0.38 ± 0.06. Relative *Cyp17a1* mRNA levels (*n* = 5): control, 1.00 ± 0.11; 3 h, 29.24 ± 5.22; 6 h, 28.16 ± 3.66; 12 h, 8.68 ± 2.94; 24 h, 1.96 ± 0.77. Asterisks indicate statistically significant differences compared with the no-treatment control (*P < 0.05; **P < 0.01; ***P < 0.001; ****P < 0.0001).

cAMP is a key mediator in the LH-induced synthesis of testosterone in Leydig cells. We therefore examined the effects of 8-Br-cAMP, a membrane-permeable cAMP analog, on the expression of steroidogenic genes involved in testosterone biosynthesis over a 24 h time course in MA-10 cells (Fig. 4). After 3 h of 8-Br-cAMP stimulation, the mRNA levels of *Star* dramatically increased to ∼92-fold of the control and then gradually decreased to ∼19-fold of the control by 24 h. A great increase in *Cyp17a1* expression was also observed at 3–6 h (∼29-fold of control), after which the expression descended to near the baseline at 24 h of 8-Br-cAMP stimulation. However, the expression of *Cyp11a1* and *Hsd3b1* was slightly increased (∼1.8 fold) at 3 h of 8-Br-cAMP treatment.

**Figure 5:**
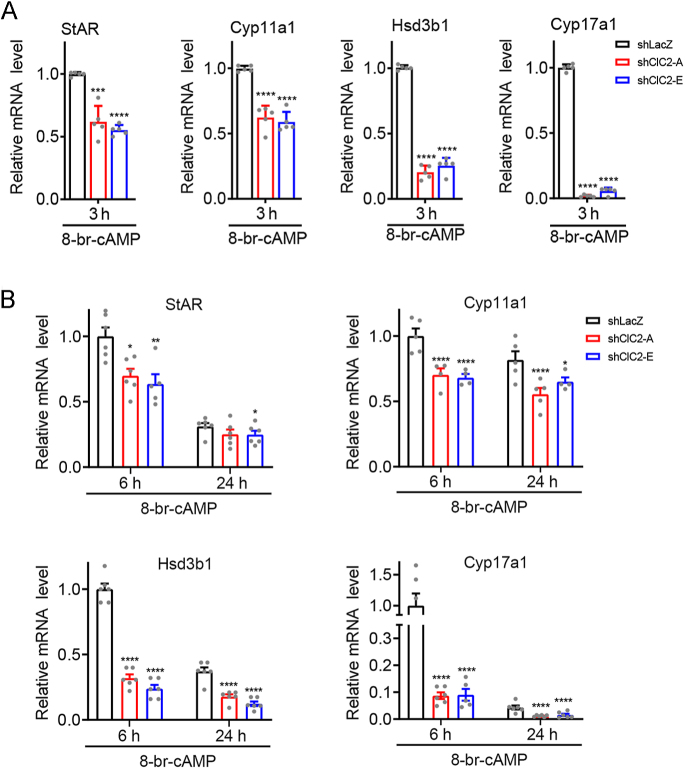
Decline in 8-Br-cAMP–stimulated expression of steroidogenic genes in Leydig cells by ClC-2 knockdown. MA-10 cells stably expressing shClC-2 or control shLacZ were subject to 0.5 mM 8-Br-cAMP treatment for the indicated durations. (A) Treatment for 3 h. The mRNA levels for the indicated steroidogenic genes were quantified by RT-qPCR, followed by normalization with respect to the corresponding shLacZ control. Relative *Star* mRNA levels (*n* = 6): shLacZ, 1.00 ± 0.02; shClC2-A, 0.62 ± 0.13; shClC2-E, 0.55 ± 0.04. Relative *Cyp11a1* mRNA levels (*n* = 4–5): shLacZ, 1.00 ± 0.02; shClC2-A, 0.62 ± 0.09; shClC2-E, 0.59 ± 0.08. Relative *Hsd3b1* mRNA levels (*n* = 5–6): shLacZ, 1.00 ± 0.02; shClC2-A, 0.20 ± 0.05; shClC2-E, 0.25 ± 0.06. Relative *Cyp17a1* mRNA levels (*n* = 5–6): shLacZ, 1.00 ± 0.03; shClC2-A, 0.02 ± 0.01; shClC2-E, 0.06 ± 0.03. (B) Treatment for 6 or 24 h. The mRNA levels for the indicated steroidogenic genes were quantified by RT-qPCR, followed by normalization with respect to the corresponding shLacZ control at 6 h treatment. Relative *Star *mRNA levels (*n* = 6) ⇒ 6 h: shLacZ, 1.00 ± 0.07; shClC2-A, 0.70 ± 0.05; shClC2-E, 0.64 ± 0.07. 24 h: shLacZ, 0.31 ± 0.03; shClC2-A, 0.25 ± 0.04; shClC2-E, 0.25 ± 0.03. Relative *Cyp11a1* mRNA levels (*n* = 4–5) ⇒ 6 h: shLacZ, 1.00 ± 0.06; shClC2-A, 0.70 ± 0.05; shClC2-E, 0.68 ± 0.03. 24 h: shLacZ, 0.82 ± 0.07; shClC2-A, 0.55 ± 0.05; shClC2-E, 0.65 ± 0.03. Relative *Hsd3b1* mRNA levels (*n* = 5–6) ⇒ 6 h: shLacZ, 1.00 ± 0.04; shClC2-A, 0.32 ± 0.03; shClC2-E, 0.24 ± 0.03. 24 h: shLacZ, 0.37 ± 0.03; shClC2-A, 0.18 ± 0.02; shClC2-E, 0.12 ± 0.02. Relative *Cyp17a1* mRNA levels (*n* = 5–6) ⇒ 6 h: shLacZ, 1.00 ± 0.20; shClC2-A, 0.09 ± 0.01; shClC2-E, 0.09 ± 0.02. 24 h: shLacZ, 0.04 ± 0.01; shClC2-A, 0.01 ± 0.00; shClC2-E, 0.02 ± 0.00. Asterisks indicate statistically significant differences compared with the corresponding shLacZ control at the same treatment time point (*P < 0.05; **P < 0.01; ***P < 0.001; ****P < 0.0001).

**Figure 6:**
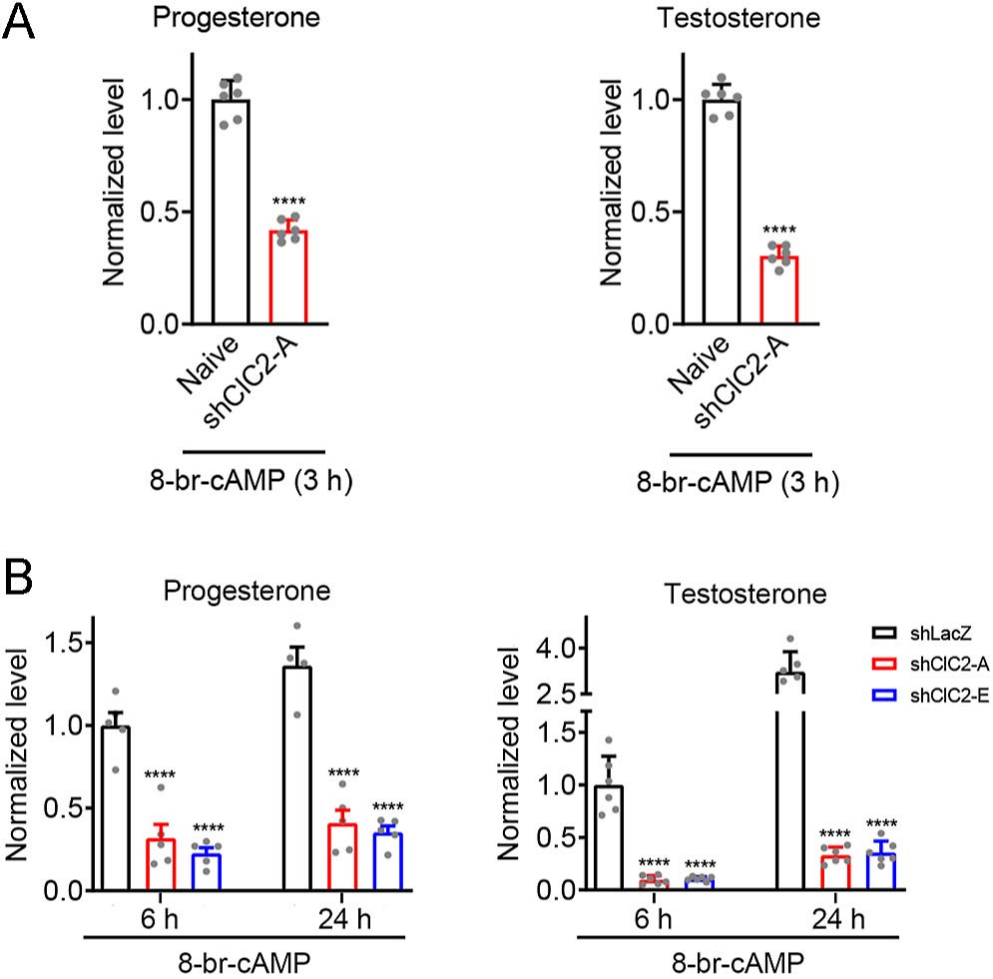
Diminution in 8-Br-cAMP–stimulated steroid production in Leydig cells by ClC-2 knockdown. (A) Naïve MA-10 cells, as well as MA-10 cells stably expressing shClC2-A, were treated with 0.5 mM 8-Br-cAMP in serum-free medium for 3 h. Steroid hormone levels in the culture medium were measured by ELISA, followed by normalization with respect to the corresponding naïve control. Normalized progesterone levels (*n* = 6): control, 1.00 ± 0.08; shClC2-A, 0.42 ± 0.05. Normalized testosterone levels (*n* = 6): control, 1.00 ± 0.07; shClC2-A, 0.30 ± 0.04. Asterisks indicate statistically significant differences compared with the corresponding naïve control (****P < 0.0001). (B) MA-10 cells stably expressing shClC-2 or control shLacZ were treated with 0.5 mM 8-Br-cAMP in serum-free medium for 6 or 24 h. The levels of progesterone or testosterone in the medium were measured by ELISA, followed by normalization with respect to the corresponding shLacZ control at 6 h treatment. Normalized progesterone levels (*n* = 5 ⇒ 6 h: shLacZ, 1.00 ± 0.08; shClC2-A, 0.32 ± 0.08; shClC2-E, 0.23 ± 0.03. 24 h: shLacZ, 1.36 ± 0.11; shClC2-A, 0.41 ± 0.08; shClC2-E, 0.35 ± 0.04. Normalized testosterone levels (*n* = 5) ⇒ 6 h: shLacZ, 1.00 ± 0.11; shClC2-A, 0.10 ± 0.02; shClC2-E, 0.11 ± 0.01. 24 h: shLacZ, 3.23 ± 0.27; shClC2-A, 0.33 ± 0.03; shClC2-E, 0.36 ± 0.04. Asterisks indicate statistically significant differences compared with the corresponding shLacZ control at the same treatment time point (****P < 0.0001).

We then determined the impact of ClC-2 knockdown on testosterone biosynthesis in cAMP-stimulated Leydig cells. In 8-Br-cAMP–treated shClC-2 MA-10 cells, the mRNA expression of the four steroidogenic genes was significantly lower than that in the control shLacZ MA-10 cells (Fig. 5). The secretion levels of progesterone and testosterone after 8-Br-cAMP treatment were also lower in shClC-2 MA-10 cells compared with those in shLacZ MA-10 cells (Fig. 6). Together, these results imply that ClC-2 may be essential for both basal and cAMP-stimulated steroidogenesis in Leydig cells.

### Contribution of T-type voltage-gated calcium channel to steroidogenesis in Leydig cells

**Figure 7:**
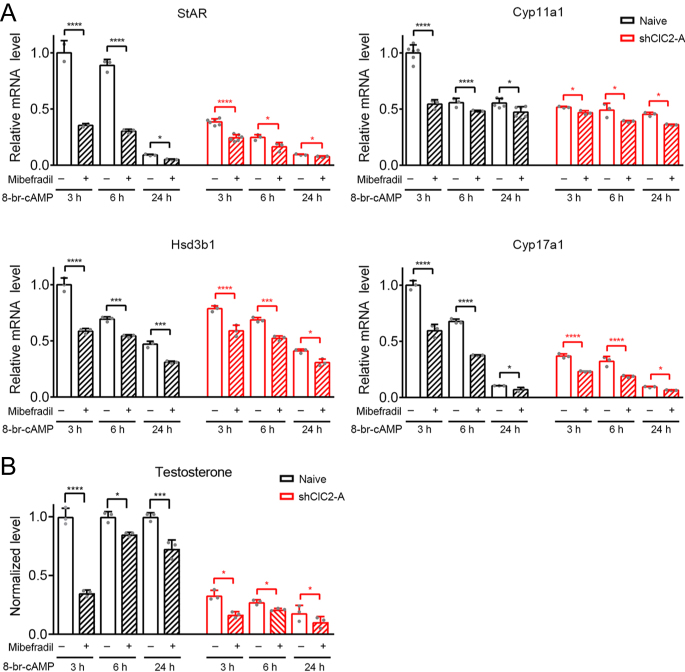
Attenuation of steroidogenic gene expression and testosterone production in MA-10 cells by the T-type voltage-gated calcium channel blocker mibefradil. Naïve MA-10 cells, as well as MA-10 cells stably expressing shClC2-A, were pretreated with 0.1% DMSO (−) or 5 μM mibefradil (+) in serum-free medium for 20 min, followed by incubation in fresh serum-free medium containing 0.5 mM 8-Br-cAMP for 3, 6, or 24 h. Steroidogenic gene expression was analyzed by RT-qPCR (A), and testosterone levels in the culture medium were measured by ELISA (B). mRNA and testosterone levels were normalized with respect to the corresponding DMSO control at 3 h treatment. (A) Relative *Star* mRNA levels (*n* = 3–6) ⇒ DMSO: 3 h, 1.00 ± 0.11; 6 h, 0.89 ± 0.05; 24 h, 0.09 ± 0.01. Mibefradil: 3 h, 0.35 ± 0.02; 6 h, 0.30 ± 0.02; 24 h, 0.05 ± 0.00. DMSO + shClC2-A: 3 h, 0.39 ± 0.03; 6 h, 0.25 ± 0.03; 24 h, 0.09 ± 0.01. Mibefradil + shClC2-A: 3 h, 0.24 ± 0.03; 6 h, 0.16 ± 0.04; 24 h, 0.08 ± 0.01. Relative *Cyp11a1* mRNA levels (*n* = 3–6) ⇒ DMSO: 3 h, 1.00 ± 0.07; 6 h, 0.56 ± 0.04; 24 h, 0.55 ± 0.04. Mibefradil: 3 h, 0.54 ± 0.04; 6 h, 0.48 ± 0.01; 24 h, 0.47 ± 0.05. DMSO + shClC2-A: 3 h, 0.52 ± 0.01; 6 h, 0.49 ± 0.06; 24 h, 0.45 ± 0.02. Mibefradil + shClC2-A: 3 h, 0.47 ± 0.02; 6 h, 0.39 ± 0.01; 24 h, 0.36 ± 0.00. Relative *Hsd3b1* mRNA levels (*n* = 3) ⇒ DMSO: 3 h, 1.00 ± 0.06; 6 h, 0.69 ± 0.02; 24 h, 0.47 ± 0.03. Mibefradil: 3 h, 0.59 ± 0.02; 6 h, 0.54 ± 0.01; 24 h, 0.31 ± 0.01. DMSO + shClC2-A: 3 h, 0.79 ± 0.03; 6 h, 0.69 ± 0.02; 24 h, 0.41 ± 0.02. Mibefradil + shClC2-A: 3 h, 0.59 ± 0.05; 6 h, 0.52 ± 0.02; 24 h, 0.31 ± 0.03. Relative *Cyp17a1* mRNA levels (*n* = 3) ⇒ DMSO: 3 h, 1.00 ± 0.04; 6 h, 0.68 ± 0.02; 24 h, 0.10 ± 0.00. Mibefradil: 3 h, 0.59 ± 0.06; 6 h, 0.38 ± 0.00; 24 h, 0.07 ± 0.02. DMSO + shClC2-A: 3 h, 0.37 ± 0.02; 6 h, 0.32 ± 0.04; 24 h, 0.09 ± 0.01. Mibefradil + shClC2-A: 3 h, 0.23 ± 0.01; 6 h, 0.19 ± 0.01; 24 h, 0.06 ± 0.00. (B) Normalized testosterone levels (*n* = 3) ⇒ DMSO: 3 h, 1.00 ± 0.07; 6 h, 1.00 ± 0.04; 24 h, 1.00 ± 0.03. Mibefradil: 3 h, 0.35 ± 0.03; 6 h, 0.85 ± 0.02; 24 h, 0.73 ± 0.08. DMSO + shClC2-A: 3 h, 0.33 ± 0.04; 6 h, 0.27 ± 0.02; 24 h, 0.18 ± 0.07. Mibefradil + shClC2-A: 3 h, 0.17 ± 0.03; 6 h, 0.21 ± 0.01; 24 h, 0.10 ± 0.05. Asterisks indicate statistically significant differences compared with the corresponding DMSO control (*P < 0.05, **P < 0.01, ***P < 0.001, ****P < 0.0001).

LH-induced testosterone production in Leydig cells involves calcium influx via Ca^2+^ channels, such as the T-type voltage-gated calcium channel (Costa & Varanda 2007, Costa *et al.* 2010, Lee *et al.* 2010, Costa *et al.* 2011). It is unclear, however, whether the T-type voltage-gated calcium channel may play a similar role in MA-10 cells as well. To address this issue, we evaluated the effect of the T-type calcium channel blocker mibefradil on 8-Br-cAMP–stimulated (3, 6, or 24 h) steroidogenesis in MA-10 cells. As depicted in Fig. 7A, pre-treatment with 5 μM mibefradil substantially reduced the effect of 8-Br-cAMP on *Star*, *Cyp11a1*, *Hsd3b1*, and *Cyp17a1* mRNA levels in naïve MA-10 cells. Likewise, 8-Br-cAMP–stimulated testosterone production was notably decreased in the presence of mibefradil (Fig. 7B). In shClC-2 MA-10 cells, where testosterone synthesis was significantly diminished, pre-treatment with mibefradil also attenuated 8-Br-cAMP–stimulated steroidogenesis (Fig. 7A and B). Collectively, these observations are consistent with the idea that the T-type voltage-gated calcium channel, as well as the ClC-2 chloride channel, critically contributes to cAMP-stimulated testosterone production in MA-10 cells.

### Enhanced ClC-2 protein expression in Leydig cells promotes testosterone synthesis

**Figure 8:**
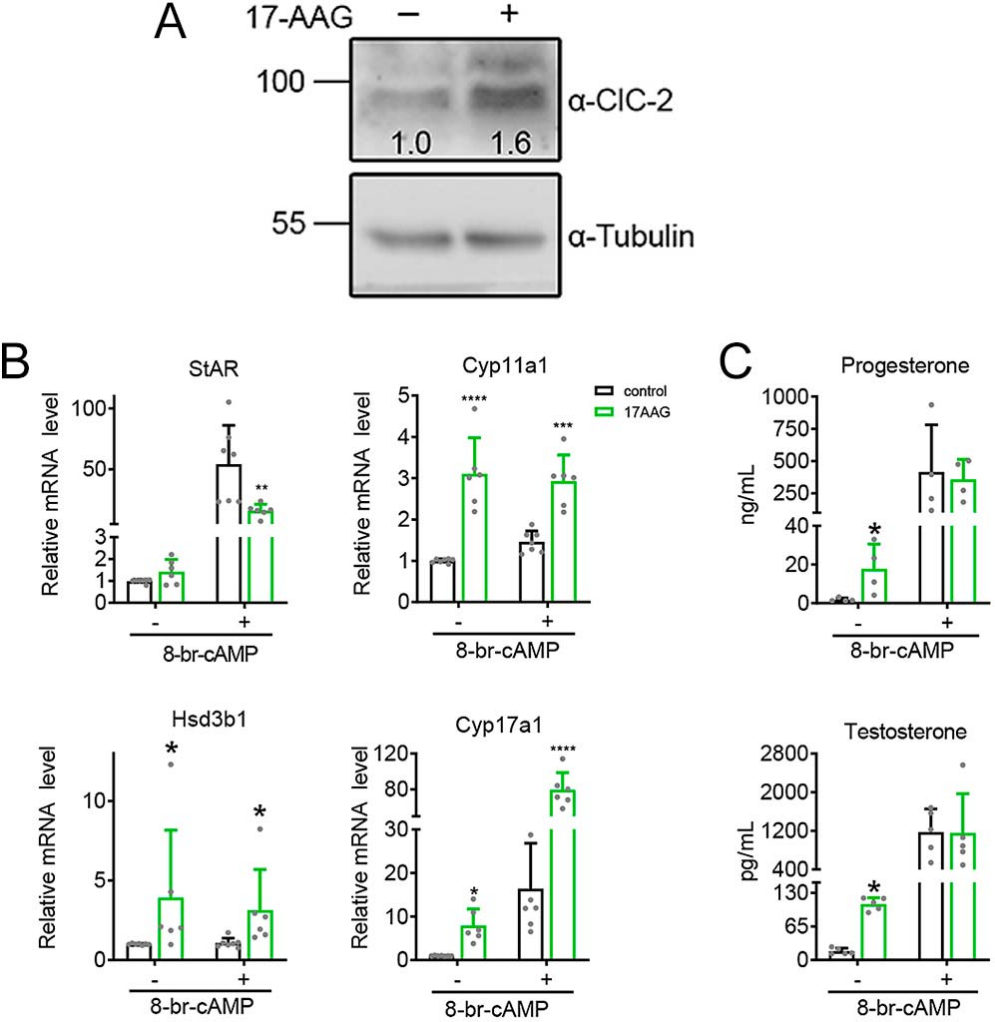
Enhancement of ClC-2 protein level, steroidogenic gene expression, and steroid production in Leydig cells by 17-AAG. (A) MA-10 cells were treated with 1 μM 17-AAG for 24 h. Treatment with 0.1% DMSO was employed as the control. The original uncropped immunoblots are presented in Supplementary Fig. 3. (B and C) MA-10 cells were treated with 1 μM 17-AAG for 18 h, followed by treatment with or without 0.5 mM 8-Br-cAMP for 6 h. The mRNA levels for the indicated steroidogenic genes were quantified using RT-qPCR (B), and the levels of progesterone or testosterone in the medium were measured by ELISA (C). mRNA and hormone levels were normalized with respect to the corresponding DMSO control. (B) Relative *Star* mRNA levels: (*n* = 5) ⇒ Without 8-Br-cAMP: DMSO, 1.00 ± 0.09; 17-AAG, 1.42 ± 0.56. With 8-Br-cAMP: DMSO, 54.24 ± 31.81; 17-AAG, 16.01 ± 5.32. Relative *Cyp11a1* mRNA levels (*n* = 6–7) ⇒ without 8-Br-cAMP: DMSO, 1.00 ± 0.05; 17-AAG, 3.11 ± 0.87. With 8-Br-cAMP: DMSO, 1.46 ± 0.26; 17-AAG, 2.93 ± 0.63. Relative *Hsd3b1* mRNA levels (*n* = 6–7) ⇒ Without 8-Br-cAMP: DMSO, 1.00 ± 0.04; 17-AAG, 3.93 ± 4.25. With 8-Br-cAMP: DMSO, 1.08 ± 0.31; 17-AAG, 3.14 ± 2.57. Relative *Cyp17a1* mRNA levels (*n* = 6–7) ⇒ Without 8-Br-cAMP: DMSO, 1.00 ± 0.07; 17-AAG, 7.91 ± 3.80. With 8-Br-cAMP: DMSO, 16.39 ± 10.45; 17-AAG, 79.49 ± 19.23. (C) Progesterone levels: (*n* = 4) ⇒ without 8-Br-cAMP: DMSO, 1.01 ± 0.92; 17-AAG, 17.81 ± 12.82. With 8-Br-cAMP: DMSO, 414.59 ± 366.79; 17-AAG, 357.59 ± 155.81. Testosterone levels (*n* = 5) ⇒ without 8-Br-cAMP: DMSO, 16.88 ± 6.10; 17-AAG, 108.34 ± 12.36. With 8-Br-cAMP: DMSO, 1,172.74 ± 476.76; 17-AAG, 1,155.58 ± 814.14. Asterisks indicate statistically significant differences compared with the corresponding DMSO control (*P < 0.05, **P < 0.01, ***P < 0.001, ****P < 0.0001).

We previously found that 17-AAG, a heat shock protein 90 (Hsp90) inhibitor, promoted endogenous ClC-2 protein expression in MA-10 cells (Fig. 8A; Supplementary Fig. 3) (Fu *et al.* 2021). We therefore went on to determine the potential effects of 17-AAG on steroidogenesis in MA-10 cells. Figure 8B shows that treatment of MA-10 cells with 17-AAG increased mRNA expression of *Cyp11a1*, *Hsd3b1*, and *Cyp17a1*, but not *Star*, under basal and 8-Br-cAMP stimulation conditions. The basal secretion of progesterone and testosterone was also increased in MA-10 cells with 17-AAG treatment (Fig. 8C). These results imply that 17-AAG can facilitate testosterone production, probably via increasing ClC-2 protein levels in Leydig cells. However, 17-AAG failed to detectably enhance the promoting effect of 8-Br-cAMP stimulation on progesterone and testosterone levels in MA-10 cells (Fig. 8C), suggesting that steroid hormone production might have reached a plateau state in 8-Br-cAMP–treated MA-10 cells.

### Reduced testosterone levels in the testes of mice injected with shClC-2

**Figure 9:**
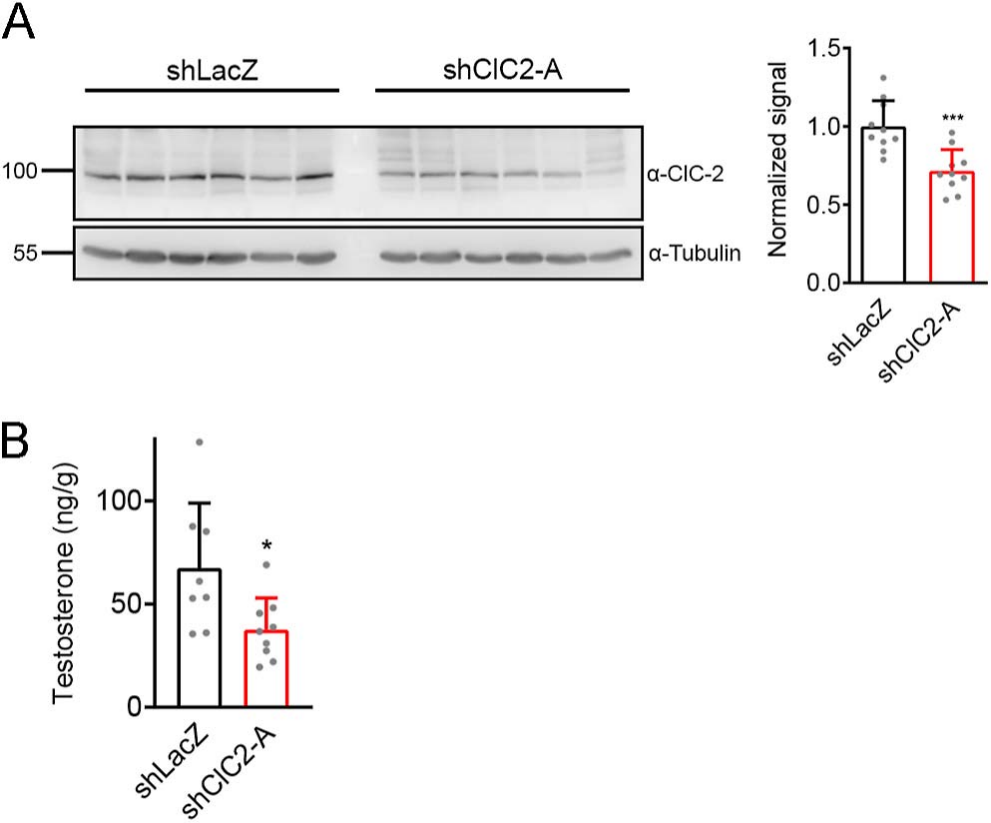
Reduction in ClC-2 protein and testosterone levels in mice subject to intratesticular injection of shClC2. The testes of 2-month-old mice were injected intratesticularly with a lentivirus delivering either shClC2 or shLacZ. The testes were harvested 7 days post-injection. (A) ClC-2 protein levels were quantified by standardizing with the corresponding α-tubulin levels, followed by normalization with respect to the corresponding shLacZ control. Relative ClC-2 protein levels (*n* = 10): shLacZ, 1.00 ± 0.16; shClC2-A, 0.72 ± 0.14. (B) Testosterone levels were measured by ELISA (*n* = 9–10): shLacZ, 67.50 ± 31.41; shClC2-A, 37.59 ± 15.35. Asterisks indicate statistically significant differences compared with the corresponding shLacZ control (*P < 0.05; ***P < 0.001). The original uncropped immunoblots are presented in Supplementary Fig. 4.

To further investigate the role of ClC-2 in regulating testosterone production in the testes, we directly injected shRNA lentivirus into the testes of adult mice *in vivo*. The shClC-2 or control shLacZ lentivirus was administered into the interstitial space via intratesticular injection (Kojima *et al.* 2003, Penny *et al.* 2017). Previous studies have shown that Leydig cells, but not Sertoli cells or germ cells, are efficiently transduced by intratesticular injection. Seven days after lentivirus injection, the testes were harvested for western blot and testosterone analyses. Figure 9A (Supplementary Fig. 4) illustrates a significant decrease in ClC-2 protein levels in the testes of shClC-2–injected mice compared with those in the control mice. Importantly, the intratesticular testosterone levels with shClC-2 treatment were significantly lower than those with shLacZ administration (Fig. 9B). These results validate the role of ClC-2 in the production of testosterone in the testes.

## Discussion

In line with the previous identification of severe testicular degeneration and male infertility in *Clcn2* knockout mice (Bosl *et al.* 2001, Nehrke *et al.* 2002), in the present study, we showed that ClC-2 is expressed in Leydig cells. shRNA knockdown of ClC-2 resulted in significantly decreased testosterone levels and reduced expression of androgen biosynthetic genes in Leydig MA-10 cells. In addition, *in vivo* intratesticular injection of an shClC-2 lentivirus led to a reduction in the levels of ClC-2 protein and testosterone in the testes of adult mice. To the best of our knowledge, we have presented the first direct evidence indicating that ClC-2 chloride channels critically regulate testosterone biosynthesis by modulating steroidogenic gene expression in Leydig cells.

The steroidogenic function of Leydig cells is primarily regulated by LH from the pituitary gland. LH binds to its G protein-coupled receptor, activating adenylate cyclase, which increases cAMP production and subsequently activates the cAMP-dependent protein kinase A (PKA) pathway (Zirkin & Papadopoulos 2018). cAMP/PKA is the main signaling mediator for regulating testosterone biosynthesis and gene expression. Chloride channels have been identified in Leydig cells and play a role in LH-mediated steroidogenesis. Noulin and Joffre utilized patch-clamp techniques to show that rat Leydig cells exhibit a hyperpolarization-activated chloride conductance, which is influenced by cAMP (Noulin & Joffre 1993*a*,*b*). In rat Leydig cells, the production of testosterone induced by low levels of LH is inhibited by the chloride channel blocker SITS (Choi & Cooke 1990). Furthermore, removal of extracellular chloride enhanced progesterone production and the expression of STAR protein in MA-10 cells in response to cAMP stimulation (Ramnath *et al.* 1997). Consistent with these observations, we showed in this study that ClC-2 crucially affects testosterone production in Leydig MA-10 cells.

Recent studies have reported that gain-of-function mutations in *CLCN2* are linked to hyperaldosteronism (Fernandes-Rosa *et al.* 2018, Scholl *et al.* 2018), wherein enhanced ClC-2 channel function leads to cell depolarization and subsequent activation of voltage-gated calcium channels in the adrenal zona glomerulosa. Activation of these channels, in turn, increases the intracellular calcium concentration and thereby promotes expression of steroidogenic genes and the synthesis of aldosterone. In Leydig cells, LH may also exert its steroidogenic effects through increasing intracellular calcium levels (Sullivan & Cooke 1986, Kumar *et al.* 1994, Rossato *et al.* 2001). Several studies revealed that the calcium signaling pathway is necessary for cAMP-induced *Star* gene expression and steroid synthesis in Leydig cells (Martin *et al.* 2008, Abdou *et al.* 2013). Furthermore, activation of voltage-gated calcium channels has been shown to be essential for steroidogenesis in Leydig cells (Manna *et al.* 1999, Costa & Varanda 2007, Costa *et al.* 2010, Lee *et al.* 2010, Costa *et al.* 2011). Herein, our data support the idea that the ClC-2 chloride channel, as well as the T-type voltage-gated calcium channel (Fig. 7), critically contributes to cAMP-stimulated testosterone production in Leydig MA-10 cells. We therefore propose that, in Leydig cells, ClC-2 channels may facilitate chloride efflux and thereby induce significant membrane depolarization to activate voltage-gated calcium channels, which would elevate intracellular calcium levels and enhance testosterone production via increased expression of steroidogenic genes.

In mouse testes, ClC-2 expression has been observed in Sertoli cells and Leydig cells, but not in germ cells (Bosl *et al.* 2001, Fu *et al.* 2020, Goppner *et al.* 2021). ClC-2 is believed to regulate the ionic environment in seminiferous tubules (Bosl *et al.* 2001), and its roles in Sertoli cells have been investigated using cell-specific *Clcn2* knockout mice (Goppner *et al.* 2021). A targeted disruption of *Clcn2* in Sertoli cells results in testicular degeneration and a loss of male germ cells, resembling the phenotype observed in *Clcn2* knockout mice. Thus, ClC-2 is crucial for Sertoli cells to support normal spermatogenesis. However, the functional significance of ClC-2 in Leydig cells was not thoroughly explored. The primary function of Leydig cells, regulated by LH, is the production of testosterone. A previous study has found that serum testosterone levels in *Clcn2* knockout mice are 2.2 times lower than those in normal mice, although this difference was not statistically significant (Bosl *et al.* 2001). Our findings in MA-10 Leydig cells indicate that knockdown of ClC-2 significantly decreased basal and 8-Br-cAMP–stimulated testosterone production (Figs 3, 6, 7). Transcriptional regulation of genes related to steroidogenesis is a crucial step in controlling steroid hormone biosynthesis. We further showed that ClC-2 downregulation resulted in a reduction in the expression of steroidogenic genes, including *Star*, *Cyp11a1*, *Hsd3b1,* and *Cyp17a1* (Figs 2 and 5). ClC-2 knockdown had the most prominent impact on *Cyp17a1* expression, reducing it by 80–90%, which is critical since CYP17A1 is a vital enzyme necessary for androgen production (Yoshimoto & Auchus 2015). In contrast, the role of CYP17A1 in the biosynthesis of aldosterone and cortisol is substantially less important, which might explain why *Clcn2* knockout mice failed to exhibit detectable alteration in steroid hormone production in the adrenal gland.

It is well known in both rats and humans that testosterone levels in the testes are much higher than those found in serum; therefore, intratesticular testosterone is essential for supporting spermatogenesis (Jarow & Zirkin 2005, Ramaswamy & Weinbauer 2014, Walker 2021). Our *in vivo* study further showed that reduced ClC-2 expression in Leydig cells of mouse testes led to a decrease in testicular testosterone levels (Fig. 9). This observation may support the previous report indicating that ClC-2 deficiency leads to reduced serum testosterone levels in male mice (Bosl *et al.* 2001). Moreover, in light of the previously identified association of human *CLCN2* gene mutations with male reproductive disorders (Di Bella *et al.* 2014, Jeworutzki *et al.* 2021), our findings provide strong evidence suggesting that, in these patients, loss-of-function mutations in the ClC-2 chloride channel result in testosterone deficiency, which may be mechanistically linked to their clinical manifestation of azoospermia and infertility.

In conclusion, our data highlight a prominent role of ClC-2 chloride channels in the regulation of testicular steroidogenesis in Leydig cells. ClC-2 deficiency may significantly downregulate the expression of steroidogenic genes in Leydig cells, thereby disrupting the physiological control of testosterone production by the pituitary gland. Testosterone deficiency may therefore be one of the key pathophysiological mechanisms underlying male infertility associated with ClC-2 mutation.

## Supplementary materials



## Declaration of interest

The authors declare that there is no conflict of interest that could be perceived as prejudicing the impartiality of the work reported.

## Funding

This work was supported by grants from the National Science and Technology Council, Taiwan, to CJJ (113-2314-B-A49-008, 112-2314-B-A49-069), CYT (112-2314-B-002-028, 111-2314-B-002-045, 110-2314-B-002-238, 111-2320-B-002-017-MY3), and MCH (109-2320-B-002-015-MY3), as well as the Ministry of Education, Taiwan, to MCH (NTU-JP-112L7238). CJJ also received grant support from the Ministry of Education, Taiwan, for the Featured Areas Research Center Program within the framework of the Higher Education Sprout Project in the Brain Research Center, National Yang Ming Chiao Tung University.

## Author contribution statement

SJF, MSS, CYT, and CYH performed biochemical and morphological experiments in cells and animals. SJF, MSS, and MCH carried out data analyses and figure preparation. CJJ, CYT, and MCH conceived the study, as well as contributed to research design and data interpretation. SJF, MSS, and MCH drafted and revised the manuscript with input from the coauthors. All authors approved the final version of the manuscript.

## References

[bib1] Abdou HS, Villeneuve G & Tremblay JJ 2013 The calcium signaling pathway regulates leydig cell steroidogenesis through a transcriptional cascade involving the nuclear receptor NR4A1 and the steroidogenic acute regulatory protein. Endocrinology 154 511–520. (10.1210/en.2012-1767) 23183170

[bib2] Ascoli M 1981 Characterization of several clonal lines of cultured Leydig tumor cells: gonadotropin receptors and steroidogenic responses. Endocrinology 108 88–95. (10.1210/endo-108-1-88) 6257492

[bib3] Bi MM, Hong S, Zhou HY, et al. 2014 Chloride channelopathies of ClC-2. Int J Mol Sci 15 218–249. (10.3390/ijms15010218) PMC3907807 24378849

[bib4] Blanz J, Schweizer M, Auberson M, et al. 2007 Leukoencephalopathy upon disruption of the chloride channel ClC-2. J Neurosci 27 6581–6589. (10.1523/jneurosci.0338-07.2007) 17567819 PMC6672451

[bib5] Bosl MR, Stein V, Hubner C, et al. 2001 Male germ cells and photoreceptors, both dependent on close cell-cell interactions, degenerate upon ClC-2 Cl(-) channel disruption. Embo J 20 1289–1299. (10.1093/emboj/20.6.1289) 11250895 PMC145530

[bib6] Chang C, Chen YT, Yeh SD, et al. 2004 Infertility with defective spermatogenesis and hypotestosteronemia in male mice lacking the androgen receptor in Sertoli cells. Proc Natl Acad Sci U S A 101 6876–6881. (10.1073/pnas.0307306101) 15107499 PMC406435

[bib7] Cheng CY & Mruk DD 2012 The blood-testis barrier and its implications for male contraception. Pharmacol Rev 64 16–64. (10.1124/pr.110.002790) 22039149 PMC3250082

[bib8] Choi MS & Cooke BA 1990 Evidence for two independent pathways in the stimulation of steroidogenesis by luteinizing hormone involving chloride channels and cyclic AMP. FEBS Lett 261 402–404. (10.1016/0014-5793(90)80602-f) 2155829

[bib9] Clark BJ, Wells J, King SR, et al. 1994 The purification, cloning, and expression of a novel luteinizing hormone-induced mitochondrial protein in MA-10 mouse Leydig tumor cells. Characterization of the steroidogenic acute regulatory protein (StAR). J Biol Chem 269 28314–28322. (10.1016/s0021-9258(18)46930-x) 7961770

[bib10] Cooke BA, Ashford L, Abayasekara DR, et al. 1999 The role of chloride ions in the regulation of steroidogenesis in rat Leydig cells and adrenal cells. J Steroid Biochem Mol Biol 69 359–365. (10.1016/s0960-0760(99)00076-x) 10419013

[bib11] Costa RR & Varanda WA 2007 Intracellular calcium changes in mice Leydig cells are dependent on calcium entry through T-type calcium channels. J Physiol 585 339–349. (10.1113/jphysiol.2007.137950) 17932157 PMC2375479

[bib12] Costa RR, Varanda WA & Franci CR 2010 A calcium-induced calcium release mechanism supports luteinizing hormone-induced testosterone secretion in mouse Leydig cells. Am J Physiol Cell Physiol 299 C316–C323. (10.1152/ajpcell.00521.2009) 20519450

[bib13] Costa RR, Reis RI, Aguiar JF, et al. 2011 Luteinizing hormone (LH) acts through PKA and PKC to modulate T-type calcium currents and intracellular calcium transients in mice Leydig cells. Cell Calcium 49 191–199. (10.1016/j.ceca.2011.02.003) 21367452

[bib14] De Gendt K, Swinnen JV, Saunders PT, et al. 2004 A Sertoli cell-selective knockout of the androgen receptor causes spermatogenic arrest in meiosis. Proc Natl Acad Sci U S A 101 1327–1332. (10.1073/pnas.0308114100) 14745012 PMC337052

[bib15] de Mattos K, Pierre KJ & Tremblay JJ 2023 Hormones and signaling pathways involved in the stimulation of leydig cell steroidogenesis. Endocrines 4 573–594. (10.3390/endocrines4030041)

[bib16] Depienne C, Bugiani M, Dupuits C, et al. 2013 Brain white matter oedema due to ClC-2 chloride channel deficiency: an observational analytical study. Lancet Neurol 12 659–668. (10.1016/s1474-4422(13)70053-x) 23707145

[bib17] Di Bella D, Pareyson D, Savoiardo M, et al. 2014 Subclinical leukodystrophy and infertility in a man with a novel homozygous CLCN2 mutation. Neurology 83 1217–1218. (10.1212/wnl.0000000000000812) 25128180

[bib18] Edwards MM, Marin de Evsikova C, Collin GB, et al. 2010 Photoreceptor degeneration, azoospermia, leukoencephalopathy, and abnormal RPE cell function in mice expressing an early stop mutation in CLCN2. Investig Ophthalmol Vis Sci 51 3264–3272. (10.1167/iovs.09-4887) 20071672 PMC2891478

[bib19] Fernandes-Rosa FL, Daniil G, Orozco IJ, et al. 2018 A gain-of-function mutation in the CLCN2 chloride channel gene causes primary aldosteronism. Nat Genet 50 355–361. (10.1038/s41588-018-0053-8) 29403012

[bib20] Fu SJ, Hu MC, Peng YJ, et al. 2020 CUL4-DDB1-CRBN E3 Ubiquitin Ligase regulates proteostasis of ClC-2 chloride channels: implication for aldosteronism and leukodystrophy. Cells 9 1332. (10.3390/cells9061332) 32466489 PMC7348978

[bib21] Fu SJ, Hu MC, Hsiao CT, et al. 2021 Regulation of ClC-2 chloride channel proteostasis by molecular chaperones: correction of leukodystrophy-associated defect. Int J Mol Sci 22 5859. (10.3390/ijms22115859) 34070744 PMC8197790

[bib22] Goppner C, Soria AH, Hoegg-Beiler MB, et al. 2021 Cellular basis of ClC-2 Cl(-) channel-related brain and testis pathologies. J Biol Chem 296 100074. (10.1074/jbc.ra120.016031) 33187987 PMC7949093

[bib23] Jarow JP & Zirkin BR 2005 The androgen microenvironment of the human testis and hormonal control of spermatogenesis. Ann N Y Acad Sci 1061 208–220. (10.1196/annals.1336.023) 16467270

[bib24] Jeworutzki E, Tuttelmann F, Rothenberg I, et al. 2021 Can unlikely neanderthal chloride channel CLC-2 gene variants provide insights in modern human infertility? Cell Physiol Biochem 55 301–310. (10.33594/000000376) 34148308

[bib25] Jeyaraj DA, Grossman G & Petrusz P 2005 Altered bioavailability of testosterone in androgen-binding protein-transgenic mice. Steroids 70 704–714. (10.1016/j.steroids.2005.03.015) 15939447

[bib26] Kojima Y, Sasaki S, Umemoto Y, et al. 2003 Effects of adenovirus mediated gene transfer to mouse testis in vivo on spermatogenesis and next generation. J Urol 170 2109–2114. (10.1097/01.ju.0000092898.91658.08) 14532865

[bib27] Kumar S, Blumberg DL, Canas JA, et al. 1994 Human chorionic gonadotropin (hCG) increases cytosolic free calcium in adult rat Leydig cells. Cell Calcium 15 349–355. (10.1016/0143-4160(94)90010-8) 8033193

[bib28] Lee JH, Kim JU, Kim C, et al. 2010 Inhibitory actions of mibefradil on steroidogenesis in mouse Leydig cells: involvement of Ca(2+) entry via the T-type Ca(2+) channel. Asian J Androl 12 807–813. (10.1038/aja.2010.51) 20694017 PMC3739071

[bib29] Manna PR, Pakarinen P, El-Hefnawy T, et al. 1999 Functional assessment of the calcium messenger system in cultured mouse Leydig tumor cells: regulation of human chorionic gonadotropin-induced expression of the steroidogenic acute regulatory protein. Endocrinology 140 1739–1751. (10.1210/en.140.4.1739) 10098511

[bib30] Martin LJ, Boucher N, Brousseau C, et al. 2008 The orphan nuclear receptor NUR77 regulates hormone-induced StAR transcription in Leydig cells through cooperation with Ca2+/calmodulin-dependent protein kinase I. Mol Endocrinol 22 2021–2037. (10.1210/me.2007-0370) 18599618 PMC5419457

[bib31] Mellon SH & Vaisse C 1989 cAMP regulates P450scc gene expression by a cycloheximide-insensitive mechanism in cultured mouse Leydig MA-10 cells. Proc Natl Acad Sci U S A 86 7775–7779. (10.1073/pnas.86.20.7775) 2554289 PMC298153

[bib32] Miller WL & Auchus RJ 2011 The molecular biology, biochemistry, and physiology of human steroidogenesis and its disorders. Endocr Rev 32 81–151. (10.1210/er.2010-0013) 21051590 PMC3365799

[bib33] Mruk DD & Cheng CY 2015 The Mammalian blood-testis barrier: its biology and regulation. Endocr Rev 36 564–591. (10.1210/er.2014-1101) 26357922 PMC4591527

[bib34] Nehrke K, Arreola J, Nguyen HV, et al. 2002 Loss of hyperpolarization-activated Cl(-) current in salivary acinar cells from Clcn2 knockout mice. J Biol Chem 277 23604–23611. (10.1074/jbc.m202900200) 11976342

[bib35] Ni FD, Hao SL & Yang WX 2019 Multiple signaling pathways in Sertoli cells: recent findings in spermatogenesis. Cell Death Dis 10 541. (10.1038/s41419-019-1782-z) 31316051 PMC6637205

[bib36] Noulin JF & Joffre M 1993a Characterization and cyclic AMP-dependence of a hyperpolarization-activated chloride conductance in Leydig cells from mature rat testis. J Membr Biol 133 1–15. (10.1007/bf00231873) 8391581

[bib37] Noulin JF & Joffre M 1993b Cyclic AMP- and calcium-activated chloride currents in Leydig cells isolated from mature rat testis. Arch Int Physiol Biochim Biophys 101 35–41. (10.3109/13813459308998126) 7684276

[bib38] Penny GM, Cochran RB, Pihlajoki M, et al. 2017 Probing GATA factor function in mouse Leydig cells via testicular injection of adenoviral vectors. Reproduction 154 455–467. (10.1530/rep-17-0311) 28710293 PMC5589507

[bib39] Ramaswamy S & Weinbauer GF 2014 Endocrine control of spermatogenesis: role of FSH and LH/testosterone. Spermatogenesis 4 e996025. (10.1080/21565562.2014.996025) 26413400 PMC4581062

[bib40] Ramnath HI, Peterson S, Michael AE, et al. 1997 Modulation of steroidogenesis by chloride ions in MA-10 mouse tumor Leydig cells: roles of calcium, protein synthesis, and the steroidogenic acute regulatory protein. Endocrinology 138 2308–2314. (10.1210/en.138.6.2308) 9165016

[bib41] Rossato M, Nogara A, Merico M, et al. 2001 Store-operated calcium influx and stimulation of steroidogenesis in rat leydig cells: role of Ca2+-activated K+ channels. Endocrinology 142 3865–3872. (10.1210/en.142.9.3865) 11517164

[bib42] Saez JM 1994 Leydig cells: endocrine, paracrine, and autocrine regulation. Endocr Rev 15 574–626. (10.1210/edrv-15-5-574) 7843069

[bib43] Scholl UI, Stolting G, Schewe J, et al. 2018 CLCN2 chloride channel mutations in familial hyperaldosteronism type II. Nat Genet 50 349–354. (10.1038/s41588-018-0048-5) 29403011 PMC5862758

[bib44] Simard J, Ricketts ML, Gingras S, et al. 2005 Molecular biology of the 3β-hydroxysteroid dehydrogenase/Δ5-Δ4 isomerase gene family. Endocr Rev 26 525–582. (10.1210/er.2002-0050) 15632317

[bib45] Smith LB & Walker WH 2014 The regulation of spermatogenesis by androgens. Semin Cell Dev Biol 30 2–13. (10.1016/j.semcdb.2014.02.012) 24598768 PMC4043871

[bib46] Sullivan MHF & Cooke BA 1986 The role of Ca2+ in steroidogenesis in Leydig cells. Stimulation of intracellular free Ca2+ by lutropin (LH), luliberin (LHRH) agonist and cyclic AMP. Biochem J 236 45–51. (10.1042/bj2360045) 3024621 PMC1146784

[bib47] Walker WH 2021 Androgen actions in the testis and the regulation of spermatogenesis. In Molecular Mechanisms in Spermatogenesis, pp 175–203. Eds CY Cheng & F Sun. Cham: Springer International Publishing. (10.1007/978-3-030-77779-1_9) 34453737

[bib48] Willems A, Batlouni SR, Esnal A, et al. 2010 Selective ablation of the androgen receptor in mouse sertoli cells affects sertoli cell maturation, barrier formation and cytoskeletal development. PLoS One 5 e14168. (10.1371/journal.pone.0014168) 21152390 PMC2994754

[bib49] Xiao X, Mruk DD, Wong CK, et al. 2014 Germ cell transport across the seminiferous epithelium during spermatogenesis. Physiology 29 286–298. (10.1152/physiol.00001.2014) 24985332 PMC4103058

[bib50] Yoshimoto FK & Auchus RJ 2015 The diverse chemistry of cytochrome P450 17A1 (P450c17, CYP17A1). J Steroid Biochem Mol Biol 151 52–65. (10.1016/j.jsbmb.2014.11.026) 25482340 PMC4456341

[bib51] Zirkin BR & Papadopoulos V 2018 Leydig cells: formation, function, and regulation. Biol Reprod 99 101–111. (10.1093/biolre/ioy059) 29566165 PMC6044347

