# Supplementary material for: Regulation of testosterone synthesis in Leydig cells by ClC-2 chloride channel
Source: Reproduction. 2025 Jul 18;170(2):e240432. doi: 10.1530/REP-24-0432 (PMC12278444; doi:10.1530/REP-24-0432)

Supplementary Figure 1. Additional immunofluorescence micrographs related to Figure 1A.

(A) Immunofluorescence staining of ClC-2 (green) and Hsd3b-1 (red; a specific marker for Leydig cells) was performed on mouse testicular sections. Nuclei were stained with DAPI (blue). St, seminiferous tubules; Lc, Leydig cells. Scale bar, 25 μm. (B) Corresponding original uncropped and unadjusted images.

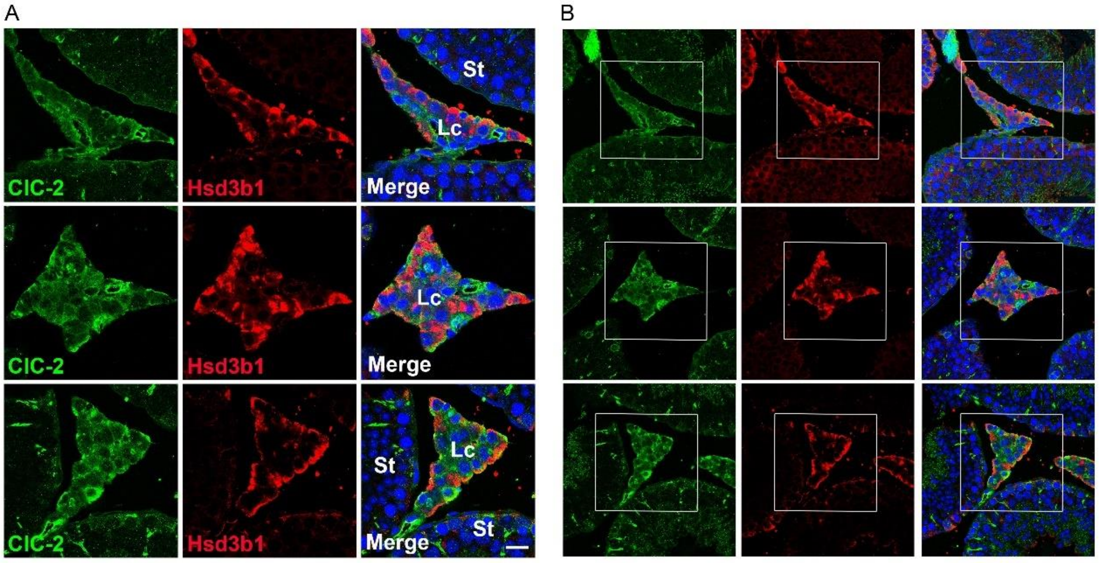

Supplementary Figure 2. The original, uncropped, and unadjusted images for Figure 1.

(A) For the immunofluorescence micrographs shown in Figure 1A. (B) For the immunofluorescence micrographs shown in Figure 1B. (C) For the immunoblots shown in Figure 1C. (D) For the immunoblots shown in Figure 1D.

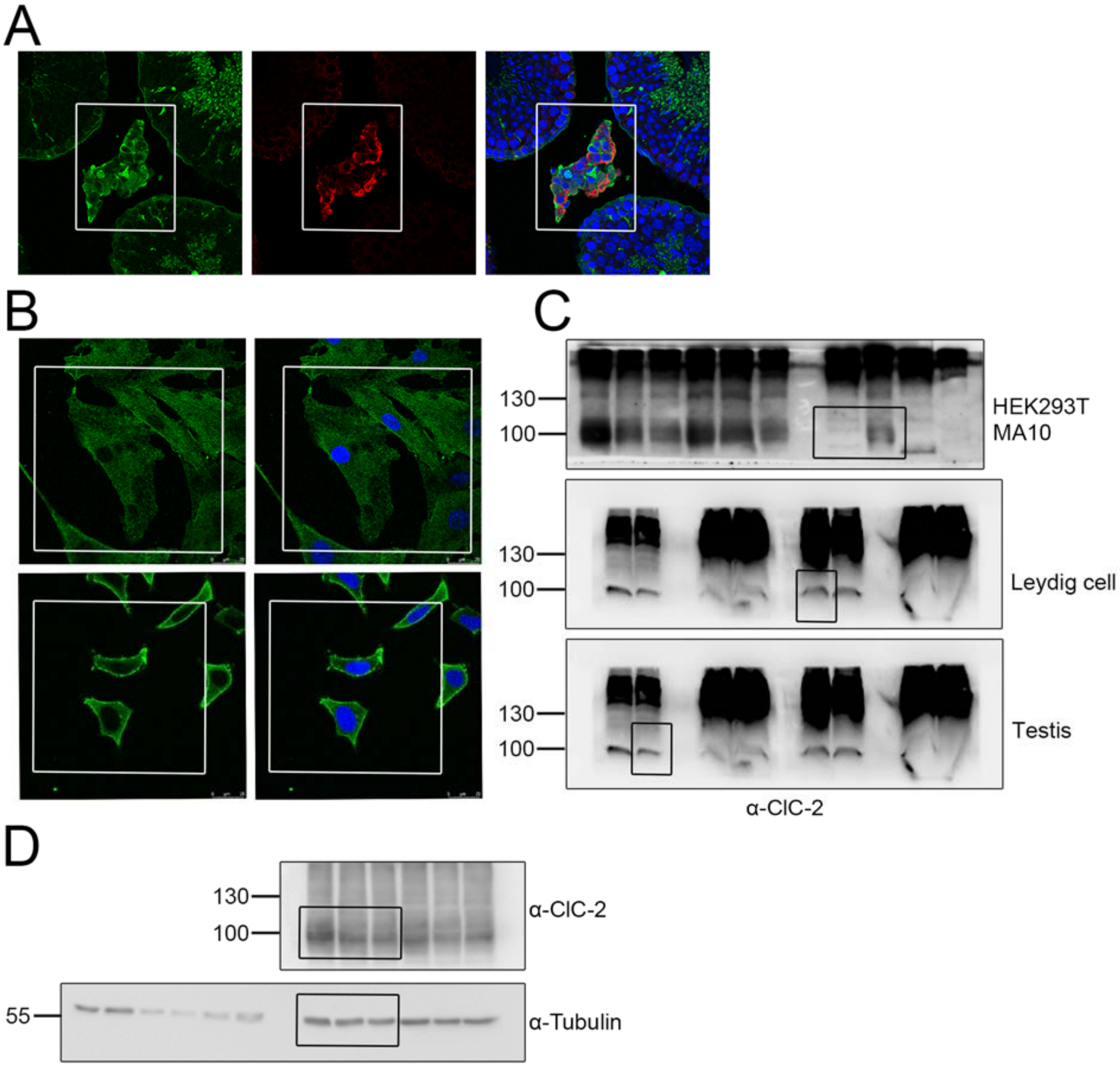

Supplementary Figure 3. The original, uncropped, and unadjusted images for Figure 8.

For the immunoblots shown in Figure 8A.

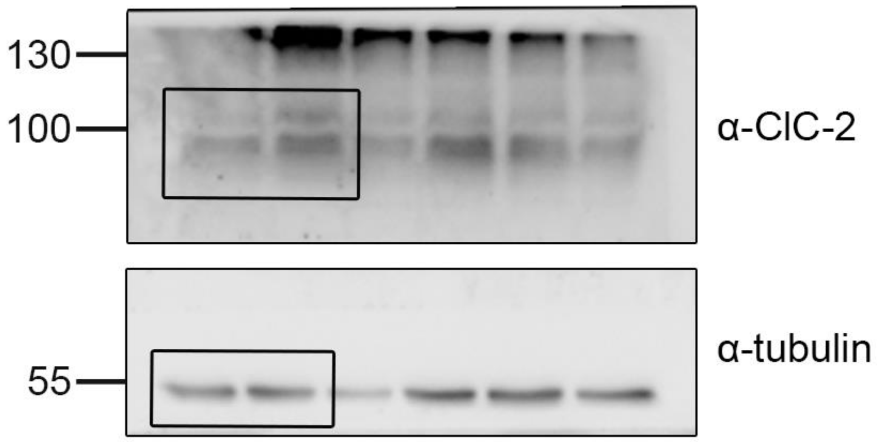

Supplementary Figure 4. The original, uncropped, and unadjusted images for Figure 9.

For the immunoblots shown in Figure 9A.

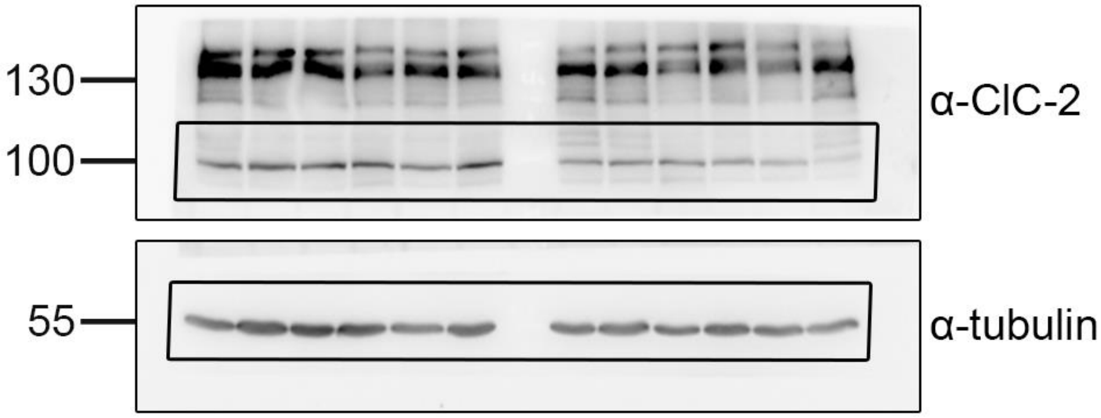

Supplement: Supplementary file 1 [file supplementary_materials.pdf]
